# Advancements in Treatment Strategies for Chronic Mesenteric Ischemia: A Comprehensive Review

**DOI:** 10.3390/jcm12227112

**Published:** 2023-11-15

**Authors:** Genti Xhepa, Andrea Vanzulli, Lucilla Violetta Sciacqua, Agostino Inzerillo, Pierre Faerber, Anna Maria Ierardi, Gianpaolo Carrafiello, Filippo Del Grande, Alexis Ricoeur

**Affiliations:** 1 Istituto Di Imaging ella Svizzera Italiana (IIMSI), Clinica Di Radiologia, Ente Ospedaliero Cantonale (EOC), 6900 Lugano, Switzerland; filippo.delgrande@eoc.ch; 2 Interventional Radiology Unit, University Hospital of Geneva (HUG), 1205 Geneva, Switzerland; agostino.inzerillo@hcuge.ch (A.I.); pierre.faerber@hcuge.ch (P.F.); alexis.ricoeur@hcuge.ch (A.R.); 3 Residency Program in Diagnostic and Interventional Radiology, Università degli Studi di Milano, 20126 Milan, Italy; andrea.vanzulli@unimi.it (A.V.); lucilla.sciacqua@unimi.it (L.V.S.); 4 Department of Radiology, Foundation IRCCS Ca’ Granda-Ospedale Maggiore Policlinico, University of Milan, 20122 Milan, Italy; anna.ierardi@unimi.it; 5 Diagnostic and Interventional Radiology Department, IRCCS Ca’ Granda Fondazione Ospedale Maggiore Policlinico, Università degli Studi di Milano, 20126 Milan, Italy; gianpaolo.carrafiello@unimi.it; 6 Facoltà Di Scienze Biomediche, Campus Est, Università Della Svizzera Italiana (USI), 6900 Lugano, Switzerland

**Keywords:** review, chronic mesenteric ischemia, superior mesenteric artery, revascularization, treatment, endovascular revascularization (ER), percutaneous transluminal angioplasty (PTA), primary stenting (PMAS)

## Abstract

Chronic mesenteric ischemia (CMI) arises from the inability to achieve adequate intestinal blood flow after meals, leading to an imbalance between oxygen and metabolite supply and demand. The true incidence of CMI remains uncertain. However, the occurrence of mesenteric artery occlusive disease (MAOD) is relatively common among the elderly population. Delays in diagnosing CMI can often be attributed to several factors, including the variability in patient symptoms and the range of potential causes for chronic abdominal pain with weight loss. Mikkelson pioneered the introduction of a surgical treatment for occlusive lesions of the superior mesenteric artery (SMA) in 1957. The inaugural performance of endovascular revascularization (ER) for visceral vessels took place in 1980. The literature has documented two types of endovascular revascularization (ER) methods: percutaneous transluminal angioplasty (PTA) and primary stenting (PMAS). Despite the limited quality of available evidence, the consensus among experts is strongly in favor of PMAS over PTA alone for the treatment of atherosclerotic mesenteric artery stenosis. There are several key areas of focus for chronic mesenteric ischemia (CMI) treatment. Randomized controlled trials comparing different stent types, such as covered stents versus bare metal stents, are needed to evaluate efficacy, patency rates, and long-term outcomes in CMI patients.

## 1. Introduction

In 1869, Chienne [1] was the first to identify and describe chronic mesenteric ischemia (CMI) resulting from inadequate blood supply in the arteries. Building upon this knowledge, Councilman [2] provided a detailed anatomical account of occlusions in the celiac trunk (CT) and superior mesenteric artery (SMA) in 1894. Then, in 1936, Dunphy [3] made the significant observation that abdominal angina serves as a vascular disorder, indicating a potential precursor to fatal occlusion in the intestinal blood vessels.

CMI arises from the inability to achieve adequate intestinal blood flow after meals, leading to an imbalance between oxygen and metabolite supply and demand. This hemodynamic disturbance is typically the result of atherosclerotic occlusive disease occurring at the entrances of the mesenteric vessels, such as the celiac artery (CA), superior mesenteric artery (SMA), and inferior mesenteric artery (IMA), which accounts for 35–75% of cases [4,5]. Other authors stated that atherosclerotic narrowing of the mesenteric arteries is responsible for more than 95% of cases of mesenteric arterial stenosis (MAS) [6,7,8].

Furthermore, nonatherosclerotic causes include vasculitis, fibromuscular dysplasia, segmental arterial mediolysis, and median arcuate ligament syndrome [4].

The occurrence of mesenteric artery occlusive disease (MAOD) is relatively common among the elderly population, particularly among individuals with occlusive disease observed in other vascular areas. In a prospective study conducted by Wilson et al. [8], involving over 500 patients, it was reported that 17% of elderly adults exhibited significant occlusive disease in the CA or SMA. Despite this, no deaths attributable to mesenteric infarction were recorded during the follow-up period (mean duration of 6.5 years) among this population. Additionally, none of the surviving participants (71%) reported symptoms consistent with chronic mesenteric ischemia (CMI) [8].

Notably, unlike most other atherosclerotic diseases, chronic mesenteric ischemia is observed more frequently in women [9]. This discrepancy is probably due to differences in the orientation of the mesenteric vessels in relation to the aorta, as women tend to have a more acute angle with the aorta compared to men [10,11].

The diagnosis of CMI relies on a combination of specific clinical symptoms and the presence of hemodynamically significant MAOD. While the classic symptoms of CMI typically include postprandial abdominal pain, weight loss, and food fear, it is important to acknowledge that this triad may not always be present, even at the time of revascularization. Instead, patients may present with nonspecific gastrointestinal complaints such as abdominal discomfort, nausea/vomiting, diarrhea, and constipation [10,12,13,14,15].

The abdominal pain experienced by CMI patients is commonly described as mid-abdominal, crampy, or dull. It typically arises within 30 min after eating and can persist for up to 6 h, coinciding with the postprandial hemodynamic changes. In a series of patients who underwent mesenteric revascularization, abdominal pain was present in 96% of cases but only occurred after eating in 74% of patients. Weight loss was observed in 84% of cases, while food fear was reported by 45% [10].

Notably, there was a significant delay of approximately 15 months from the onset of symptoms to the definitive diagnosis in this series [10].

Another series from Europe found that the typical postprandial abdominal pain was present in 85% of patients with CMI, accompanied by weight loss in 77% and food fear in 63%. Additionally, 56% of patients experienced symptoms such as diarrhea, nausea, or vomiting [16].

Malnutrition has been found to have a notable impact on the outcomes of patients undergoing revascularization for CMI. Allain et al. [17] conducted a study involving 54 CMI patients who underwent revascularization and assessed their nutritional status using parameters such as body mass index, percentage of weight loss, and serum albumin levels. They discovered that 70% of the patients were malnourished, and this condition was associated with increased perioperative mortality and decreased long-term survival. Interestingly, not all patients with CMI are cachectic or malnourished at the time of presentation [17].

In a recent series reported by Mansukhani et al., 35% of the patients undergoing revascularization for CMI were overweight or obese, with a body mass index exceeding 25. The authors attributed this trend to the obesity epidemic across the country [18].

When comparing the prevalence rates of various vascular diseases, it becomes evident that CMI has a significantly lower prevalence. The estimated prevalence for peripheral artery disease (PAD) ranges from 3% to 10%, while coronary artery disease (CAD) has a prevalence of 4.5% and cardiovascular disease (CVD) has a prevalence of 2.4% [7,19]. In contrast, CMI has a prevalence of only 0.03% (30 per 100,000 individuals), indicating its rarity. The relatively low prevalence of CMI can be attributed to the presence of an abundant collateral circulation in the gastrointestinal (GI) tract. This collateral circulation is a remnant of the intricate embryonal network, which provides the GI tract with a remarkable level of flexibility to prevent ischemia caused by stenoses in most cases. This adaptive mechanism plays a crucial role in maintaining adequate blood supply to the GI tract and contributes to the lower incidence of CMI compared to other vascular conditions [7,10]. In the reported cohort by van Noord et al., there were no significant differences in the clinical presentation among patients with single-vessel CMI and multivessel CMI. However, there is one exception to this finding: in patients with end-stage multivessel CMI, the clinical presentation tends to deviate from the typical pattern. They experience more continuous pain, longer duration of postprandial pain, and are more likely to exhibit symptoms such as lack of energy and diarrhea. This atypical presentation is attributed to severely diminished blood flow, which is insufficient to sustain basal metabolism. These patients are at an increased risk of developing acute bowel infarction and are now referred to as having acute-on-chronic mesenteric ischemia (A-o-CMI) [7].

### 1.1. Visceral Artery Anatomy and Function

**Figure 1:**
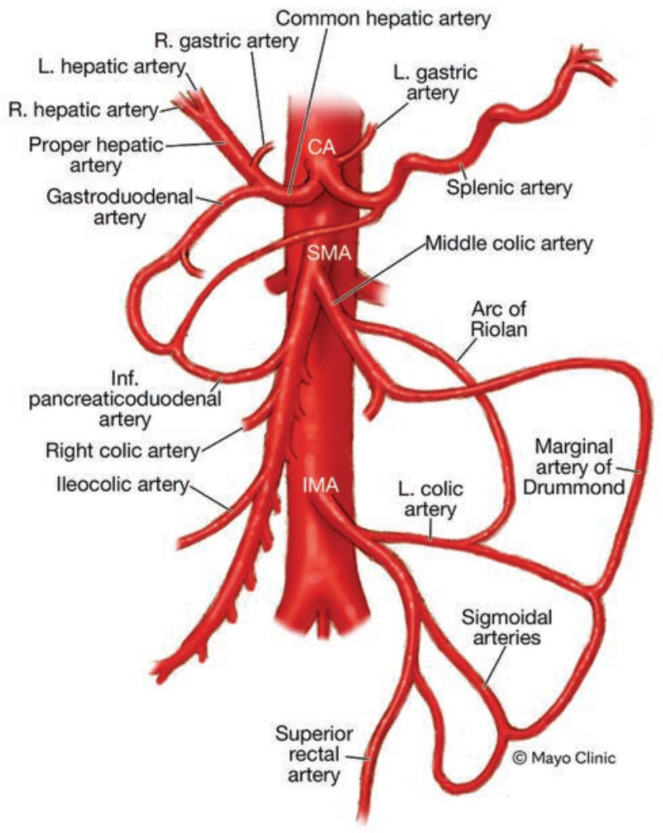
A representation of typical arterial blood supply in the mesenteric arteries and highlights common alternative routes. Abbreviations used: R. = right, L. = left, CA = celiac artery, SMA = superior mesenteric artery, Inf. = inferior, IMA = inferior mesenteric artery.

The blood supply to the gastrointestinal tract is primarily facilitated by three main arteries: CA, SMA, and IMA. Additionally, collateral circulation plays a significant role in ensuring blood flow to the GI tract. The internal iliac artery, specifically the hemorrhoidal arteries, contributes significantly to these collateral connections. There are specific arterial connections that bridge the CA and SMA, including the gastroduodenal and pancreaticoduodenal arteries. These arteries establish crucial connections between the two main arteries, facilitating blood flow to the relevant regions. Furthermore, the marginal artery of Drummond and the arc of Riolan, which is a meandering or central anastomotic artery located near the inferior mesenteric vein in the mesentery, serve as important connections between the IMA (via the left colic artery) and the SMA (via the middle colic artery). Figure 1 highlights the mesenteric arterial vascularization and the principal anastomoses [20].

These intricate collateral networks ensure adequate blood supply and maintain vascular flexibility within the gastrointestinal tract [21,22].

Under fasting conditions, the gastrointestinal tract receives approximately 20% of the total blood flow [23].

In a study conducted by Someya et al., it was demonstrated that the baseline blood flow in the CA increased after meals. The mean blood flow in the CA rose from 450 mL/min at baseline to 700 mL/min, reaching its peak within 10 min after the meal. Similarly, the SMA exhibited an increase in blood flow from a mean baseline of 400 mL/min to over 800 mL/min, with the peak occurring after approximately 40 min [24]. This heightened blood flow in the SMA can persist for up to 3–6 h [25,26].

The magnitude and duration of the postprandial increase in blood flow depend on the volume and composition of the meal. Studies have shown that meals containing higher amounts of fat elicit the largest and longest-lasting postprandial peaks in blood flow [27]. On the other hand, protein-rich meals induce the smallest increase in blood flow, while carbohydrate-rich meals fall in between fat- and protein-induced responses [28].

The hyperemic response, characterized by increased blood flow, begins with the anticipation of a meal. However, the most prominent hemodynamic effects are observed after the ingestion of food and its movement into the small bowel. Vasodilation of the mesenteric vessels typically initiates 3 to 5 min after ingestion and can persist for 4 to 6 h, depending on the composition of the meal. The peak response is usually observed within 30 to 90 min [5]. The SMA plays a crucial role in the increased blood flow during the postprandial period, as evidenced by the significant increase in end-diastolic velocity (EDV) observed on Doppler ultrasound (DUS) imaging during a meal challenge [29]. Within the layers of the bowel wall, the mucosa receives a greater quantity of blood flow compared to the submucosa or muscularis. This makes the mucosa layer more susceptible to ischemic damage if the blood supply is compromised [28]. In patients with CMI, the postprandial hyperemic response is impaired or reduced due to the occlusion or stenosis of the mesenteric vessels. This diminished response can lead to an imbalance between the oxygen and metabolite supply and demand, resulting in the cardinal symptoms of CMI: pain (related to visceral nerves), malabsorption (affecting the intestinal mucosa), and altered bowel emptying (influencing peristalsis) [30,31,32].

### 1.2. Natural History of Mesenteric Ischemia

Delays in diagnosing CMI can often be attributed to several factors, including the variability in patient symptoms, the broad range of potential causes for chronic abdominal pain with weight loss, and the relatively low prevalence of CMI in the general population. These delays commonly result from extensive diagnostic investigations conducted by primary care physicians or gastroenterologists before referring the patient to a vascular specialist [5].

Given the lack of reliable functional tests for diagnosing CMI, the diagnosis heavily relies on a combination of the appropriate clinical presentation and the presence of MAOD. Harki et al. [33] attempted to develop a predictive model utilizing clinical and anatomical findings to enhance diagnostic accuracy. They identified several factors as potential diagnostic predictors, including female sex, weight loss, presence of cardiovascular disease, duration of symptoms, and the presence of stenosis in the SMA or CA. In a study involving 666 patients, Van Dijk et al. [34] prospectively validated this predictive model. The model demonstrated good discrimination, particularly when an additional predictor for the cause of CA stenosis was included. Importantly, 94% of high-risk patients identified by the model had sustained symptom relief after revascularization, confirming the diagnosis of CMI. Nevertheless, only 8% of low-risk patients had confirmed CMI [34].

The understanding of the natural progression of CMI has largely been shaped by studying the clinical course of patients before they undergo intervention, which is often influenced by the duration of symptoms or the occurrence of acute mesenteric ischemia (AMI). It is important to acknowledge that these studies are subject to significant selection bias. Without treatment, patients with CMI can experience severe weight loss and malnutrition, leading to wasting away. Additionally, they may progress to the more acute form of AMI. Reports suggest that the 5-year mortality rate for untreated CMI patients approaches 86% [6]. Interestingly, up to 50% of patients with AMI present with thrombosis of an existing lesion and exhibit prior symptoms consistent with CMI [35]. In contrast, revascularization procedures, whether open surgeries or endovascular interventions, have demonstrated excellent outcomes in terms of relieving symptoms and achieving long-term graft or mesenteric vessel patency [12,16,36,37]. However, it is worth noting that there can be a considerable delay in the diagnosis of CMI, with the average time from initial presentation to diagnosis spanning 15 to 35 months [7,10,38,39,40]. However, there is one exception to this finding: in patients with end-stage multivessel CMI, the clinical presentation tends to deviate from the typical pattern. They experience more continuous pain, longer duration of postprandial pain, and are more likely to exhibit symptoms such as lack of energy and diarrhea. This atypical presentation is attributed to severely diminished blood flow, which is insufficient to sustain basal metabolism. These patients are at an increased risk of developing acute bowel infarction and are now referred to as having acute-on-chronic mesenteric ischemia (A-o-CMI) [7].

In a contemporary meta-analysis encompassing 78 studies spanning the years from 1956 to 2020, the aggregation of data reveals an overall mortality rate of 59.6% (95% CI 55.5 to 63.6). A sub-analysis of 24 studies exclusively focusing on patients who underwent revascularization procedures demonstrates a notably lower mortality rate of 33.9%. Furthermore, an examination of 27 studies that reported on the short-term outcomes of mesenteric venous thrombosis (MVT) reveals a pooled mortality rate of 24.6% [41].

Revascularization may have a role in the treatment of patients displaying symptoms consistent with CMI and experiencing occlusive disease that affects only one mesenteric vessel, specifically the SMA. The conventional belief has been that CMI manifests when at least two out of the three mesenteric vessels are affected, benefiting from the collateral network and redundancy in the mesenteric circulation. However, this teaching may not be entirely accurate. It is indeed possible to develop CMI solely through the involvement of a single mesenteric vessel, typically resulting from an inadequate collateral network between these vessels. In practical medical settings, it is not uncommon to come across patients presenting with vague abdominal symptoms, who are subsequently diagnosed with significant occlusive disease localized to the SMA or CA.

### 1.3. Diagnostic Workflow for CMI

Patients with CMI may exhibit abdominal pain and weight loss, but the differential diagnosis is broad, with gastrointestinal malignancy being a primary concern. Unfortunately, no specific physical findings or lab tests assist in diagnosis. Endoscopy is often performed early in the evaluation for abdominal pain and weight loss, with esophagogastroduodenoscopy and colonoscopy used to rule out malignancy unless CMI is clear. Ischemic gastritis, duodenitis, and colitis findings, while nonspecific, can suggest CMI. The presence of a gastric ulcer without malignancy strongly indicates CMI. Various adjunct functional tests, such as oxygen light spectroscopy and intestinal tonometry, have been described for CMI diagnosis but lack widespread clinical utility [5].

Mesenteric Doppler ultrasound is a proficient screening modality for chronic mesenteric ischemia (CMI). While it poses technical challenges, seasoned practitioners consistently attain dependable outcomes. A peak systolic velocity (PSV) of 275 cm/s is indicative of significant stenosis in the superior mesenteric artery (SMA), demonstrating a sensitivity of 92% and specificity of 96%. Conversely, a PSV of 200 cm/s suggests stenosis in the celiac artery (CA) with a positive predictive value (PPV) of 80% and a negative predictive value (NPV) of 99%, resulting in an overall accuracy of 96%. Nevertheless, supplementary imaging is requisite to validate and meticulously delineate mesenteric arterial occlusive disease (MAOD) for surgical strategizing [5].

CTA provides superior anatomical clarity and exclusion of alternative causes of chronic abdominal pain [5]. It utilizes advanced three-dimensional techniques with centerline measurements. CTA is endorsed as the primary diagnostic tool by both the American College of Radiologists Appropriateness Criteria and the European Society of Vascular Surgery Mesenteric Guidelines, with diagnostic accuracy ranging from 95% to 100%. In a study comparing various imaging methods, CTA displayed the best image quality, highest correlation for stenosis grading, and superior accuracy [42].

Triphasic CTA offers exceptional spatial resolution for characterizing mesenteric lesions and identifying key inflow sites necessary for intervention planning. The delayed phase can also assist in the detection of other vascular pathologies, including mesenteric venous thrombosis [5,42].

MRA is a viable alternative to CTA for CMI diagnosis, offering sensitivity and specificity exceeding 95% when compared to catheter-based arteriography [43]. The American College of Radiology (ACR) guidelines support the use of MRA as an alternative modality [44]. Moreover, in a recent study by Terlow et al. [45], clinical, imaging, and functional assessments were performed on patients suspected of CMI. Cardiac-gated 2D PC-MRI measured mesenteric blood flow before and after a meal challenge in 19 patients (8 CMI, 11 non-CMI). CMI patients displayed reduced post-meal blood flow increases in the superior mesenteric artery at 30 and 40 min, along with lower total arterial flow at 40 min. Improved venous flow was observed after mesenteric artery stenting, indicating the potential diagnostic utility of meal-induced blood flow changes in CMI identification.

Catheter arteriography, once the gold standard for diagnosing MAOD, is now less common due to safer, less invasive imaging options and complications like vessel perforation and bleeding. It remains useful when other non-invasive methods are inconclusive or when planning percutaneous interventions [5].

### 1.4. Treatment Concept Evolution over the Years

Mikkelson [46] pioneered the introduction of a surgical treatment for occlusive lesions of the superior mesenteric artery (SMA) in 1957. Subsequently, Shaw and Maynard [47] provided evidence for the efficacy of surgical thromboendarterectomy of the SMA in alleviating symptoms associated with CMI through their description of the first successful procedure.

Dotter and Judkins [48] documented the inaugural instance of catheter angioplasty utilizing a coaxial system in 1964. Following this milestone, Grüntzig and Hopff [49] innovated a dilating balloon catheter, significantly enhancing the practical implementation of this procedure. Since then, percutaneous transluminal angioplasty (PTA) has achieved remarkable success across different clinical contexts [50,51,52]. The inaugural performance of endovascular revascularization (ER) for visceral vessels took place in 1980 [53].

Subsequently, two case reports have highlighted the application of PTA in the management of abdominal angina [53,54,55].

In a comprehensive analysis of national outcomes in the United States, Schermerhorn and colleagues [56] identified a sevenfold increase in the number of mesenteric interventions between 1988 and 2006. Building upon this, Zettervall et al. [57] investigated data from the National Inpatient Sample and the Centers for Disease Control and Prevention database, revealing a more than sevenfold rise in the utilization of endovascular procedures for CMI from 2000 to 2012 (0.6–4.5/million; *p* < 0.01). Additionally, Erben et al. [58] conducted a study that examined outcomes for patients (*n* = 15,475) who underwent intervention for CMI across the country, utilizing the National Inpatient Sample data from 2000 to 2014. Their findings aligned with the aforementioned trend, as 70.6% of the patients underwent endovascular treatment.

## 2. Materials and Methods

The structure of this review and the search methodology employed has been enumerated under the following headings: papers considered for this review had the following predetermined inclusion criteria: (1) patients undergoing percutaneous treatment of the superior mesenteric artery (SMA); (2) patients undergoing percutaneous treatment of the superior mesenteric artery (SMA); (3) clinical outcomes, follow-up, and complications reported; (4) full-text publications in English available; and (5) publication date between January 1990 and September 2023. A literature search was performed in October 2023 on PubMed (MEDLINE) and Google Scholar for studies which matched the eligibility criteria using the keywords “chronic mesenteric ischemia” and “open vascular repair” or “percutaneous intervention” or “percutaneous transluminal angioplasty” or “bare metal stent” or “covered stent”; every search was conducted then for every chapter of this review. An additional manual search of the bibliographies of each included study was performed to identify studies not covered by the PubMed or Google Scholar search.

## 3. Relevant Session—Current Evidence

### 3.1. Percutaneous Transluminal Angioplasty and Primary Stenting

The literature distinguishes two endovascular revascularization (ER) methods: percutaneous transluminal angioplasty (PTA) and primary stenting (PMAS) [59], with a dearth of direct comparative prospective studies. Notably, mesenteric stenoses, often located at the ostium, are prone to PTA-induced recoil [60,61,62].

Although research on ostial renal artery stenosis suggests a higher risk of recoil and recurrent stenosis [63,64], case series and expert observations indicate prevalent mesenteric artery stenoses at the vessel’s origin, frequently with substantial calcification [61,62]. Comparative data remain inconclusive. A systematic review (328 patients) favored PMAS for technical success (95% vs. 83%), but revealed similar symptom relief (91% vs. 89%) and higher restenosis rates (35% vs. 21%) [65]. In a more recent retrospective cohort study, a lower reintervention rate was reported for PTA in comparison to PMAS, although the observed difference did not achieve significance [66].

Conversely, a retrospective study by TURBA et al. [67] showed the superior efficacy of PTA for SMA stenosis, with significantly better patency rates. Notably, no significant differences were observed in CA and IMA outcomes [67].

Despite limited quality evidence, expert consensus strongly favors PMAS over PTA alone for atherosclerotic mesenteric artery stenosis [59]. This preference aligns with guidelines from the European Society for Vascular Surgery, the American College of Radiology, and the Society for Vascular Surgery [5,44,68].

Endovascular stent placement in these cases reports success rates ranging from 85% to 100%, whereas PTA for mesenteric arterial occlusions and lesions achieves comparatively lower success rates of 65% to 85% [60].

All the characteristics listed in the previous studies are summarized in Table 1.

### 3.2. Open Revascularization vs. Endovascular Revascularization

In 1958, RS Shaw from the Massachusetts General Hospital reported the first successful open revascularization (OR) for CMI through the performance of superior mesenteric artery SMA endarterectomy. Subsequently, open surgical repair became the established standard treatment for CMI. This approach encompasses various techniques such as direct reimplantation on the aorta, antegrade or retrograde bypass grafting, and transaortic endarterectomy [47].

The selection of the specific type of open reconstruction (e.g., antegrade or retrograde, single- or multiple-vessel, aortic- or iliac-based) is based on the patient’s clinical risk assessment and anatomical considerations [69].

Despite the widespread adoption of the endovascular approach, the available data supporting its use have limitations in terms of evidence-based standards. Moreover, it remains unclear whether the early benefits of the endovascular approach outweigh the reduced long-term patency rates associated with it. The absence of randomized controlled trials (RCTs) directly comparing OR and endovascular revascularization (ER) further contributes to this knowledge gap.

The most comprehensive and rigorous study examining the perioperative and long-term outcomes of patients with CMI treated with either open or endovascular approaches is the systematic review and meta-analysis conducted by Alahdab et al. [70]. This study encompassed a total of 100 observational studies, including 22 comparative and 78 noncomparative studies, which collectively involved nearly 19,000 patients. The findings of the review indicate that the perioperative complication rate was higher in the open revascularization group, with a relative risk (RR) of 2.19 (95% confidence interval [CI], 1.84–2.60). However, there was no significant difference observed in the 30-day mortality rate between the open and endovascular groups (5.5% vs. 1.4%; RR, 1.57; 95% CI, 0.84–2.93). Regarding long-term outcomes, open revascularization was associated with a lower risk of recurrence at the 3-year mark, with an RR of 0.47 (95% CI, 0.34–0.66). However, there was no significant difference in the 3-year survival rate between the open and endovascular groups (RR, 0.96; 95% CI, 0.86–1.07).

In the meta-analysis conducted by Cai et al. [71], involving 8 studies and 569 patients, no significant disparities were identified in perioperative mortality or survival rates between the open and endovascular groups. However, the endovascular group demonstrated a lower incidence of perioperative complications, albeit with a higher rate of recurrence. Conversely, Gupta et al. [72] reported in their comprehensive analysis of 1939 patients that open repair exhibited a higher rate of perioperative complications. Remarkably, despite similar perioperative mortality and survival rates, the open repair group displayed superior 5-year primary patency (odds ratio [OR], 3.8; 95% CI, 2.4–5.8; *p* < 0.001) and 5-year freedom from recurrent symptoms (OR, 4.4; 95% CI, 2.8–7.0; *p* < 0.001) compared to the endovascular group. Turning to the meta-analysis conducted by Pecoraro et al. [73], incorporating 43 studies and 1795 patients, the endovascular group exhibited lower rates of perioperative morbidity and mortality. Nevertheless, this group presented lower patency rates without a discernible difference in survival when compared to the open repair group. Meanwhile, Saedon et al. [74], who analyzed 12 studies encompassing 7365 patients, reported no significant distinctions in perioperative morbidity, perioperative mortality, or survival between the open and endovascular groups. However, they observed a notable increase in patency (odds ratio [OR], 3.57; 95% CI, 1.83–6.97; *p* = 0.0002) within the open repair group. According to Van Petersen et al. [75], ER offers the advantage of lower short-term morbidity but entails decreased long-term primary patency compared to OR. Both ER and OR exhibited similar rates of secondary patency, albeit with a higher reintervention rate after ER. In a more recent study by Menges et al. [76], encompassing a single series of 63 patients, no significant differences were observed in terms of reintervention rate (82% after OR and 73% after ER, *p* = 0.14), 30-day mortality (0.0% after ER and 4.5% after OR, *p* = 0.069), 30-day morbidity (ER 9.8% vs. OR 31.8%, *p* = 0.030), or overall survival (OR 85% vs. ER 86%; *p* = 0.35) during a mean follow-up of 26 months. However, a substantially longer length of stay was noted after OR compared to ER (14 vs. 4 days; *p* < 0.001).

The findings from meta-analyses and single series are in line with statewide and national observations regarding revascularization for CMI. Indes et al. [77] studied patient outcomes for CMI revascularization in New York from 2000 to 2006. They found that ER became more popular, increasing from 28% to 75%. A lower perioperative mortality (11.0% vs. 20.4%), fewer mesenteric complications (6.9% vs. 17.1%), and reduced organ system complications (e.g., heart, lungs, and infections) were reported in the ER group. Wolk et al. [78] observed a rise in ER percentage from 0% in 2008 to 82% in 2017. In-hospital mortality was 2.8% (*n* = 1) and no significant mortality differences were found between treatment groups (OR 6.7%, *n* = 1 vs. ER 0%, *n* = 0; *p* = 0.42). However, major complications were more frequent after OR than ER (7% vs. 3%, *p* < 0.02). ER led to shorter hospital stays (11 ± 10 days vs. 21 ± 11 days; *p* < 0.01). No significant differences were seen in one-year primary patency (OR 91.6% vs. ER 96.8%; not significant [n.s.]), three-year primary patency (OR 91.6% vs. ER 80.6%; n.s.), and three-year symptom-free survival (OR 62.5% vs. ER 69.4%; n.s.). The authors concluded that, while ER had comparable perioperative outcomes, it had a higher technical failure rate. Conversely, OR had excellent early and late technical success.

Based on meta-analyses, case series, and nationwide experience, evidence supports using endovascular-first in CMI patients. The approach is linked to fewer complications, shorter hospital stays, and lower costs. However, the endovascular approach has higher rates of recurrent symptoms and reinterventions. Yet, long-term survival rates are comparable to open surgery, with a slight trend towards higher perioperative mortality in open surgery. Endovascular failures and recurrent symptoms do not significantly increase the risk of death. Choosing endovascular-first allows open revascularization if needed. Table 2 provides a summary of all the traits described in the studies listed above.

Reputable organizations like the Society of Vascular Surgeon [5], European Society of Vascular Surgery [68], American College of Radiology [44], and Society of Interventional Radiologists recommend endovascular-first [60].

### 3.3. Endovascular Revascularization: Balloon-Expandable Covered Stent vs. Balloon-Expandable Bare Metal Stent

Revascularization of the SMA is considered the primary objective, while the CA and IMA are considered secondary targets if SMA revascularization is not feasible or if the clinical outcome is unsatisfactory [5]. Over the past 15 years, endovascular intervention has surpassed open procedures as the most used treatment approach for CMI [66].

Currently, there is no consensus regarding the optimal choice between balloon-expandable covered stents (CS) and balloon-expandable bare metal stents (BMS) for the treatment of CMI. However, emerging evidence suggests that CS may offer increased patency rates in CMI treatment [79,80].

The utilization of covered stents (CS) entails the establishment of a physical barricade, effectively excluding atherosclerotic plaque and endothelial components. This measure serves to curtail the development of intimal hyperplasia, which is characterized by the unwarranted thickening of the innermost arterial layer. Notably, intimal hyperplasia is a pivotal element contributing to luminal constriction or occlusion, thus substantially predisposing to restenosis—the reoccurrence of arterial narrowing following prior medical intervention [80].

A pivotal attribute of CS resides in its capacity to inhibit the migration of macrophages towards the endothelium, the innermost stratum of the arterial wall. Macrophages represent a class of immune cells intricately involved in the processes of inflammation and arterial repair. When these macrophages infiltrate the endothelium, they instigate the release of pro-inflammatory mediators, such as cytokines. This orchestrated inflammatory response triggers and perpetuates the progression of neointimal hyperplasia, a multifaceted vascular reaction to injury or intervention. It entails the proliferation of smooth muscle cells and the deposition of extracellular matrix, ultimately fostering luminal narrowing. Moreover, the incorporation of polytetrafluoroethylene (PTFE) coverage, a biomaterial integral to certain stent designs, has been observed to confer advantageous outcomes by mitigating specific complications. PTFE, distinguished by its non-adherent and biocompatible characteristics, envelops the stent and furnishes a protective shield. Consequently, this safeguarding effect is associated with a diminished risk of adverse events such as arterial disruption, encompassing phenomena like dissection or injury to the arterial wall, as well as a reduced likelihood of distal embolism—the migration of embolic material to smaller downstream blood vessels. These complications, while not uncommon, are pertinent considerations following stent deployment [80].

The available literature suggests that CS can be a favorable choice for stenting in CMI. These stents offer similar benefits to those seen in other locations and their specific features, such as high radial force, suitability for short lengths, precise deployment and minimal shortening upon expansion, make them well-suited for treating the common atherosclerotic, calcified lesions typically found at the mesenteric vessel orifice. It is important to note that both balloon-expandable and self-expanding stents have complementary roles in CMI treatment. While balloon-expandable stents are preferred in most cases, self-expanding stents may be more appropriate in specific situations, such as longer lesions in the SMA beyond the orifice, managing intraluminal dissections resulting from initial endovascular techniques, and preserving significant collateral vessels, like those associated with a high take-off of a right hepatic artery [5,81,82,83].

Schoch et al. [66] achieved 100% technical success in their mesenteric intervention study. They found a significant difference between stent types: among 77 patients with bare-metal stents (BMS), 52% required reintervention, while none of the 14 patients with covered stents (CS) needed reintervention (*p* < 0.05). CS also demonstrated significantly better patency than BMS (*p* < 0.04). However, the shorter follow-up for the CS group (mean 6.6 months) compared to the longer follow-up for the BMS group (mean 17 months) limits interpretation.

Girault et al. [83] reported impressive patency rates for CS in SMA occlusive disease. At a 2-year follow-up, primary patency was 76%, primary-assisted patency was 95%, and secondary patency was 95%.

In the extensive study by Oderich et al. [13], CS showed superiority over BMS in patients. CS-treated patients had higher rates of freedom from restenosis (92% vs. 53%; *p* = 0.003), symptom recurrence (92% vs. 50%; *p* = 0.003), and reintervention (91% vs. 56%; *p* = 0.005), along with better primary patency at 3 years (92% vs. 52%; *p* < 0.003). The subgroup requiring reintervention also had better outcomes with CS, including freedom from restenosis (89% vs. 49%; *p* < 0.04), symptom recurrence (100% vs. 64%; *p* = 0.001), and reintervention (100% vs. 72%; *p* = 0.03) at 1 year. Secondary patency rates were similar between both groups.

In an extensive study by AbuRahma et al. [84], high initial success rates were observed in 83 patients BMS (97% technical and 96% clinical success). At a mean 31-month follow-up, the primary late clinical success rate was 59%, with an in-stent stenosis rate of 51% for stenosis ≥70%. Over 1 to 5 years, freedom from late recurrent symptoms ranged from 65% to 83% and survival rates ranged from 51% to 88%. There were no significant differences in primary or assisted primary patency between stents in the SMA and CA. Goldman et al. [85] studied 54 patients with CMI treated with BMS. Among them, 29.6% underwent intervention solely targeting the CA, while 70.4% received revascularization of the SMA with or without CA intervention. In the CA-only group, 50% experienced symptom recurrence, whereas, in the SMA/CA-SMA group, 21.1% had recurrence. Patients without SMA intervention had a higher risk of symptomatic recurrence (HR: 3.2, 95% CI: 1.2–8.6, *p* = 0.016) and repeat intervention (HR: 5.5, 95% CI: 1.8–16.3, *p* = 0.001). The authors concluded that SMA revascularization is vital for achieving favorable symptom outcomes and reducing the need for repeat interventions. Rajaratnam et al. [86] reported a 4% 30-day mortality rate in their study of 45 patients with CMI. Follow-up showed varying degrees of symptom resolution, with complete resolution in 65% of patients, partial improvement in 13%, no improvement in 22%, and symptom recurrence in 6%. Bulut et al. [87] analyzed data from 141 patients with CMI treated with BMS, with a focus on the involvement of the CA and SMA. The occlusion rates were 10% for the CA and 30% for the SMA. The primary patency rates at 12 and 60 months were 77.0% and 45.0%, respectively, while the primary-assisted patency rates were 90.3% and 69.8%. There were no significant differences in primary, primary-assisted, and secondary patency between the CA and SMA cases.

Haben et al. [79] studied 150 patients with CMI. Primary patency at 1 year was 86% for CA and 81% for SMA, while at 3 years it was 66% for CA and 69% for SMA. Increased age was associated with better results in the SMA. Chronic total occlusion in the SMA had worse patency and younger patients had a higher proportion of SMA occlusion. Ostial flaring was associated with improved patency in the SMA. The authors concluded that bare-metal stents remain suitable for CMI treatment.

Awouters et al. [88] retrospectively analyzed 76 CMI patients treated percutaneously. They found a 28.8% symptom relapse rate, with an average relapse time of 14.9 months over a mean 45.5-month follow-up. Cumulative incidence estimates showed relapse-free rates of 78.9%, 72.3%, and 70.3% at two, five, and ten years, respectively. Comparing circumferential and focal stenosis in the SMA, a trend towards longer relapse-free survival was observed in the circumferential group (78.2% vs. 55.5% at five years), although it was not statistically significant (*p* = 0.063). Survival did not significantly differ between the groups (*p* = 0.64).

Altinas et al. [89] conducted a study involving 245 patients who underwent endovascular intervention, categorizing them into two groups: those with chronic mesenteric ischemia (CMI) and those with acute-on-chronic mesenteric ischemia (AoCMI). In terms of one-year and three-year survival estimates, the CMI group exhibited rates of 85% and 74%, respectively. The study highlighted that the presence of superior mesenteric artery (SMA) stenosis, rather than occlusion, significantly enhanced the likelihood of successful SMA recanalization in both the CMI and AoCMI cohorts. Notably, reintervention within the first year was necessary for 5.7% of patients and this was exclusively observed in the CMI group. Additionally, a majority of patients in both groups experienced clinical improvement. For the AoCMI group, the authors reported one-year and three-year survival estimates of 67% and 54%, respectively. Moreover, the patients in the AoCMI group had a lengthier median hospital stay.

Overall, the data suggest that CS for SMA revascularization may offer advantages in terms of patency rates, freedom from reintervention, and symptomatic outcomes in CMI. However, further studies with larger sample sizes and longer follow-up durations are needed to confirm these findings and establish more definitive conclusions.

According to the ESVS [68], routine mesenteric stenting is recommended for patients requiring endovascular treatment of CMI (Class I, Lev Evv B). However, there is ongoing controversy regarding the choice between BMS and CS for treating SMA stenosis. The Society for Vascular Surgery [5] suggests the use of balloon-expandable covered intraluminal stents for treating MAOD in CMI patients, with a Grade 2 (Weak) level of recommendation and a Quality of Evidence of C (Low). In the European guidelines for CMI [59], the expert panel could not reach a consensus on recommending the use of CS due to current uncertainties regarding their superiority.

The results of two ongoing randomized controlled trials, the Dutch study (CoBaGi) including 6 centers (NCT02428582) [90] and the French study (ESTIMEC) including 26 centers (NCT03586739) [91], comparing CS and bare metal stents, are eagerly awaited.

Table 3 shows data from comparative studies between CS and BMS.

All the characteristics listed in the previous studies are summarized in Table 4 and Table 5.

The technique known as retrograde open mesenteric stenting (ROMS) was initially documented by Milner et al. in 2004 [92]. The procedure begins with a median laparotomy to diagnose mesenteric ischemia, followed by retrograde endovascular revascularization of the superior mesenteric artery. The SMA is exposed infracolic, with its branches clamped to prevent embolization, and thrombectomy is performed as needed. If the distal SMA is heavily calcified, a longitudinal arteriotomy is required. After the endovascular procedure, the arteriotomy is closed with patch angioplasty. A retrograde angiogram is conducted using a sheath and guide wire, and a catheter and softly angled guide wire are used to cross the lesion, with translesionary passage recommended to reduce the risk of aortic dissection. If retrograde crossing is unsuccessful, an antegrade approach may be considered, with a femoral or radial/brachial approach. Following angiographic confirmation of intraluminal placement, a 4 mm pre-dilatation is performed and a covered, balloon-expandable stent (6–8 mm in diameter) is deployed at the SMA’s ostium. Additional lining may be placed distally if necessary. After an angiogram confirms successful revascularization and proper closure of the arteriotomy, intestinal resection may be required for irreversible bowel ischemia [92,93].

Oderich et al. [93] analyzed data from seven academic centers (2001–2013) on 54 patients who underwent ROMS. This involved laparotomy for mesenteric stenting, with 81% having acute mesenteric ischemia (AMI) and 19% having subacute-on-chronic mesenteric ischemia. Stenting was performed on 56 mesenteric vessels. Technical success was 98% but early AMI mortality was 45%, while subacute cases had 10% early mortality. Two-year patient survival was 43%, with 76% primary patency and 90% secondary patency. Freedom from recurrence and reintervention at two years was 72% and 74%, respectively. The authors concluded that ROMS is an option when percutaneous stenting is unsuitable but AMI mortality remains high.

Sénémaud et al. [94] conducted a cohort study with 379 patients. Of the 37 patients who underwent the retrograde open mesenteric stenting (ROMS) procedure, 89% achieved technical success. The study reported in-hospital mortality of 27%, post-operative complications of 67%, and reintervention rate of 32%. After one year, estimated overall survival was 70.1%, with estimated freedom from reintervention at 61.1%. Primary patency rate at one year was 84.54% and assisted primary patency rate was 92.4%.

Cirillo-Pen et al. [95] performed ROMS on 34 patients with different types of mesenteric ischemia. The procedure achieved 91% technical success. Stent usage included CS in 58% of cases, extended CS with bare metal self-expanding stents in 6%, and BMS in 36%. Median follow-up was 3.7 years. At 1 year, freedom from reintervention was 87%, primary patency was 70%, primary-assisted patency was 87%, and secondary patency was 97%. Symptom recurrence was 95% at 1 and 3 years. The study did not evaluate stent type superiority.

In summary, ROMS is a valuable option for certain cases of mesenteric ischemia, particularly when other methods are not suitable. While it demonstrates technical success and reasonable patency rates, there remains a need for further improvement in outcomes, particularly in reducing early mortality for AMI patients and addressing symptom recurrence.

## 4. Discussion—Future Direction

There are several key areas of focus for chronic mesenteric ischemia (CMI) treatment. Encouraging collaboration among specialists from various disciplines, such as vascular surgeons, interventional radiologists, and gastroenterologists, is essential to develop comprehensive treatment algorithms and optimize patient care through a multidisciplinary approach [5,44,60,68]. Diagnostic techniques are being refined to enhance the identification and assessment of CMI, including the utilization of non-invasive imaging modalities like computed tomography angiography (CTA) and magnetic resonance angiography (MRA) for accurate evaluation of mesenteric artery stenosis or occlusion. Advanced imaging methods such as intravascular ultrasound (IVUS) or optical coherence tomography (OCT) hold potential for improving stent placement accuracy and assessing post-procedural outcomes [96]. Advancements in endovascular technologies, particularly the development of specialized stents and drug-eluting systems, have the potential to enhance the durability and effectiveness of interventions for CMI. Tissue engineering approaches, including bioresorbable scaffolds, are being explored to stimulate tissue regeneration and provide temporary support. Surgical revascularization procedures, such as mesenteric bypass surgery, remain a consideration for cases where endovascular approaches may not be suitable or effective. Tailoring stent selection and intervention strategies based on individual patient characteristics, such as age, comorbidities, anatomical variations, and severity of mesenteric artery disease, is an avenue of investigation. This may involve the development of risk stratification models to guide personalized treatment decisions.

## 5. Conclusions

Well-designed randomized controlled trials comparing different stent types, such as covered stents versus bare metal stents, are needed to evaluate efficacy, patency rates, and long-term outcomes in CMI patients. Establishing optimal stent selection criteria and improving treatment guidelines can be achieved through such studies. Collaborative research networks involving multiple centers are being established to facilitate large-scale studies and generate robust evidence for optimizing stent interventions in CMI. Future studies should also include patient-reported outcomes and quality-of-life assessments to evaluate the impact of stent interventions on functional outcomes, symptom relief, and overall well-being in CMI patients.

## Figures and Tables

**Table 1:** Comparative studies from 2005 to 2020 between angioplasty (PTA) and primary stenting (PMAS).

	Angioplasty (PTA)	Primary Stenting (PMAS)
Landis, MS et al. (2005) [61]	- Technical success rates 100% - 45% of patients undergoing PTA required further interventions - No procedure-related complications or mortality	- Technical success rates 85% - 11% of patients undergoing PMAS required further interventions - No procedure-related complications or mortality
Kougias, P et al. (2007) [65]	- Comparable symptom relief (PTA 89% vs. PMAS 91%)	- Higher technical success rate for PMAS when compared to PTA (95% vs. 83%) - Higher restenosis rate (PMAS 35% vs. PTA 21%)
Schoch, DM et al. (2011) [66]	- At long-term follow up, no significant difference was observed between PTA and PMAS	- At long-term follow up, no significant difference was observed between PTA and PMAS
Turba, UC et al. (2012) [67]	- For SMA stenosis, PTA alone exhibited superior efficacy compared to PMAS or PTA followed by stenting (*p* < 0.031)	- The patency rates of the SMA following PTA alone were significantly better than when all stents were combined (*p* < 0.014)
Björck, M et al. (2017) [68]	- For celiac artery lesions, PTA and PMAS have a higher rate of restenosis compared to open surgical bypass - A case series shows successful results for the efficacy of angioplasty of the IMA	- Celiac stenting may be considered a “bridge” to open bypass
Pillai, AK et al. (2018) [60]	- Shorter hospital stay compared with surgical revascularization - Technical success rates 79% - Thresholds 65%	- Shorter hospital stay compared with surgical revascularization - Technical success rates 94% - Thresholds 85%

**Table 2:** Comparative study from 2009 to 2022 between open revascularization and endovascular revascularization.

	Open Revascularization (OR)	Endovascular Revascularization (ER)
Indes, JE et al. (2009) [77]	- Higher mesenteric complications (OR:17.1% vs. ER: 6.9%)	- Lower overall mortality rate (ER 11.0% vs. OR 20.4%) - Reduced organ system complications (e.g., heart, lungs, and infections)
Gupta, PK et al. (2010) [72]	- Higher rate of perioperative complications - Superior 5-year primary patency	- Similar perioperative mortality and survival rates
Van Petersen, AS et al. (2010) [75]	- Increased long-term primary patency	- Lower short-term morbidity - Similar rates of secondary patency - Higher reintervention rate compared to open therapy
Pecoraro, F et al. (2013) [73]	- No difference in survival rate	- Lower rates of perioperative morbidity and mortality - Lower patency rates
Cai, W et al. (2015) [71]	- No significant disparities in perioperative mortality or survival rates	- Lower incidence of perioperative complications - Higher rate of recurrence
Saedon, M et al. (2015) [74]	- Notable increase in patency compared to endovascular treatment	- No significant distinctions in perioperative morbidity, perioperative mortality, or survival
Alahdab, F et al. (2018) [70]	- Higher perioperative complication rate (RR 2.19) - No significant difference observed in the 30-day mortality - Higher 5-year primary patency - Higher 5-year freedom from recurrent symptoms - No significant difference in the 3-year survival rate	- Lower perioperative complications - Not durable as open revascularization with higher symptom recurrence rate at 3 years - Lower risk of recurrence at the 3-year follow-up (RR of 0.47)
Merges, AL et al. (2020) [76]	- No significant differences for reintervention rate (82% after OR and 73% after ER) - 30-day mortality (0.0% after ER and 4.5% after OR) - Longer length of stay after OR compared to ER (14 vs. 4 days).	- 30-day morbidity (ER 9.8% vs. OR 31.8%, *p* = 0.030), or overall survival (OR 85% vs. ER 86%; *p* = 0.35) during a mean follow-up of 26 months
Walk, S et al. (2022) [78]	- More frequent major complications (7% vs. 3%) - No significant differences in 1-year and 3-year primary patency and 3-year symptom-free survival	- No significant mortality differences - Shorter hospital stays (ER: 11 ± 10 days vs. OR: 21 ± 11 days) - ER had comparable perioperative outcomes but higher technical failure rate

**Table 3:** Comparative studies from 2011 to 2020 between balloon-expandable cover stent and balloon-expandable bare metal stent.

	Balloon-Expandable Covered Stent (CS)	Balloon-Expandable Bare Metal Stent (BMS)
Mwipatayi, BP et al. (2016) [80]	- CS had a significantly higher patency rate than the BMS at 18, 24, 48, and 60 months	- May be associated with a higher risk of restenosis
Schoch, DM et al. (2011) [66]	- Significantly better patency than BMS (*p* < 0.04).	- Significant difference in terms of reintervention rate: among 77 patients with bare-metal stents (BMS), 52% required reintervention, while none of the 14 patients with covered stents (CS) needed reintervention (*p* < 0.05)
Oderich, GS et al. (2013) [13]	- CS showed superiority over BMS in patients - Higher rates of freedom from restenosis (92% vs. 53%), symptom recurrence (92% vs. 50%), and reintervention (91% vs. 56%) - Better primary patency at 3 years (92% vs. 52%)	- Secondary patency rates were similar between the two groups
Haben, C et al. (2020) [79]	- Increased patency rates in CMI treatment at 3 years (92% for CSs vs. 50% for BMSs)	- Higher risk of arterial disruption and distal embolism

**Table 4:** Results from 2021 clinical studies of balloon-expandable cover stent.

	Balloon-Expandable Covered Stent (CS)
Aburahma, AF et al. (2021) [84]	At a mean 31-month follow-up, the primary late clinical success rate was 59%. Over 1 to 5 years, freedom from late recurrent symptoms ranged from 65% to 83% and survival rates ranged from 51% to 88%. Despite high initial technical success for CS stenting of SMA/CA stenoses, there is a high rate of restenosis and reintervention.
Girault A et al. (2021) [83]	In individuals with mesenteric occlusive disease (MOD), mesenteric CS yields extremely satisfactory midterm results. Primary patency was 76% and secondary patency was 95% for CS in SMA occlusive disease at a 2-year follow-up.

**Table 5:** Results of clinical studies from 2017 to 2021 of balloon-expandable bare metal stent.

	Balloon-Expandable Bare Metal Stent (BMS)
Goldman et al. (2017) [85]	Among 54 patients, 29.6% underwent intervention targeting the CA, while 70.4% received revascularization of the SMA. In the CA-only group, 50% experienced symptom recurrence. In the SMA/CA-SMA group, 21.1% had recurrence. Patients without SMA intervention had a higher risk of symptomatic recurrence and repeat intervention.
Rajaratnam, K et al. (2017) [86]	After BMS, complete symptoms resolution in 65% of patients, partial improvement in 13%, no improvement in 22%, and symptom recurrence in 6%.
Bulut, T et al. (2017) [87]	141 patients with CMI treated with BMS, with an involvement of the CA and SMA. There were no significant differences in primary, primary-assisted, and secondary patency between the CA and SMA cases.
Haben, C et al. (2020) [79]	150 patients with CMI treated with BMS showed primary patency at 1 year of 86% for CA and 81% for SMA, while at 3 years it was 66% for CA and 69% for SMA. Bare-metal stents remain suitable for CMI treatment.
Awouters, J et al. (2021) [88]	28.8% symptom relapse rate (average relapse time of 14.9 months over a mean of 45.5-month follow-up). Relapse-free rates of 78.9%, 72.3%, and 70.3% at 2, 5, and 10 years. Survival did not significantly differ between the groups.

## Data Availability

All data are available within the article.
